# Impact of the Covid-19 pandemic on perinatal mental health (Riseup-PPD-COVID-19): protocol for an international prospective cohort study

**DOI:** 10.1186/s12889-021-10330-w

**Published:** 2021-02-17

**Authors:** Emma Motrico, Rena Bina, Sara Domínguez-Salas, Vera Mateus, Yolanda Contreras-García, Mercedes Carrasco-Portiño, Erilda Ajaz, Gisele Apter, Andri Christoforou, Pelin Dikmen-Yildiz, Ethel Felice, Camellia Hancheva, Eleni Vousoura, Claire A Wilson, Rachel Buhagiar, Carmen Cadarso-Suárez, Raquel Costa, Emmanuel Devouche, Ana Ganho-Ávila, Diego Gómez-Baya, Francisco Gude, Eleni Hadjigeorgiou, Drorit Levy, Ana Osorio, María Fe Rodriguez, Sandra Saldivia, María Fernanda González, Marina Mattioli, Ana Mesquita, Riseup-PPD-COVID-19 Group

**Affiliations:** 1 Psychology Department, Universidad Loyola Andalucia, Avenida de las Universidades s/n, Dos Hermanas (Sevilla), Spain; 2 School of Social Work, Bar Ilan University, Ramat Gan, Israel; 3 Graduate Program on Developmental Disorders, Center for Biological and Health Sciences - Mackenzie Presbyterian University, São Paulo, Brazil; 4 Departamento de Obstetricia y Puericultura, Facultad de Medicina, Universidad de Concepción, Concepción, Chile; 5 Department of Education Sciences and Psychology, Beder College University, Tirana, Albania; 6 Perinatal and Child Psychiatry, Le Havre Hospital, Normandie University Rouen, Rouen, France; 7 Department of Social and Behavioral Sciences, European University Cyprus, Nicosia, Cyprus; 8 Department of Psychology, Kirklareli University, Kirklareli, Turkey; 9 University of Malta, Msida, MSD 2080 Malta; 10 Sofia University “St. Kliment Ochridski”, Sofia, Bulgaria; 11 Department of Psychology, American College of Greece, Gravias 6, 15342 Agia Paraskevi, Greece; 12 Section of Women’s Mental Health, King’s College London and South London and Maudsley NHS Foundation Trust, London, United Kingdom; 13 Department of Statistics, Mathematical Analysis and Optimization, Group of Biostatistics and Biomedical Data Science, Faculty of Medicine, University of Santiago de Compostela, Rúa San Francisco, S/N, 15895 Santiago de Compostela, Spain; 14 Unidade de Investigação em Epidemiologia (EPIUnit, UIDB/04750/2020), Instituto de Saúde Pública da Universidade do Porto (ISPUP). Rua das Taipas, 135, 4050-600 Porto, Portugal; 15 Universidade Europeia, Lisbon, Portugal; 16 Université de Paris, Laboratoire de Psychopathologie et Processus de Santé (LPPS, UR4057), Groupe Hospitalier du Havre, Paris, France; 17 Univ Coimbra, Center for Research in Neuropsychology and Cognitive Behavioral Intervention, Faculty of Psychology and Educational Sciences, 3000-115 Coimbra, Portugal; 18 Department of Social, Developmental and Educational Psychology, Universidad de Huelva, Avda. de las Fuerzas Armadas, 21007 Huelva, Spain; 19 Department of Epidemiology, University Clinical Hospital of Santiago, Research Group on Epidemiology of Common Diseases, Santiago de Compostela Health Research Institute (IDIS), Santiago de Compostela, Spain; 20 Department of Nursing, Midwifery, School of Health Science, Cyprus University of Technology, Limassol, Cyprus; 21 Faculty of Psychology, Universidad Nacional de Educación a Distancia, (UNED), Madrid, Spain; 22 Departamento de Psiquiatría y Salud Mental, Facultad de Medicina, Universidad de Concepción, Concepción, Chile; 23 Faculty of Health Sciences, National University of Entre Rios, Concepción del Uruguay, Entre Ríos, Argentina; 24 Faculty of Humanities, Arts and Social Sciences, Universidad Autónoma de Entre Ríos, Concepción del Uruguay, Entre Ríos, Argentina; 25 School of Psychology, University of Minho, Campus Gualtar, 4710-057 Braga, Portugal

**Keywords:** COVID-19, Depression, Anxiety, Post-traumatic stress disorders, Postpartum, Pregnancy, Risk factors, Coping mechanisms

## Abstract

**Background:**

Corona Virus Disease 19 (COVID-19) is a new pandemic, declared a public health emergency by the World Health Organization, which could have negative consequences for pregnant and postpartum women. The scarce evidence published to date suggests that perinatal mental health has deteriorated since the COVID-19 outbreak. However, the few studies published so far have some limitations, such as a cross-sectional design and the omission of important factors for the understanding of perinatal mental health, including governmental restriction measures and healthcare practices implemented at the maternity hospitals. Within the Riseup-PPD COST Action, a study is underway to assess the impact of COVID-19 in perinatal mental health. The primary objectives are to (1) evaluate changes in perinatal mental health outcomes; and (2) determine the risk and protective factors for perinatal mental health during the COVID-19 pandemic. Additionally, we will compare the results between the countries participating in the study.

**Methods:**

This is an international prospective cohort study, with a baseline and three follow-up assessments over a six-month period. It is being carried out in 11 European countries (Albania, Bulgaria, Cyprus, France, Greece, Israel, Malta, Portugal, Spain, Turkey, and the United Kingdom), Argentina, Brazil and Chile. The sample consists of adult pregnant and postpartum women (with infants up to 6 months of age). The assessment includes measures on COVID-19 epidemiology and public health measures (Oxford COVID-19 Government Response Tracker dataset), Coronavirus Perinatal Experiences (COPE questionnaires), psychological distress (BSI-18), depression (EPDS), anxiety (GAD-7) and post-traumatic stress symptoms (PTSD checklist for DSM-V).

**Discussion:**

This study will provide important information for understanding the impact of the COVID-19 pandemic on perinatal mental health and well-being, including the identification of potential risk and protective factors by implementing predictive models using machine learning techniques. The findings will help policymakers develop suitable guidelines and prevention strategies for perinatal mental health and contribute to designing tailored mental health interventions.

**Trial registration:**

ClinicalTrials.gov Identifier: NCT04595123.

## Background

Corona Virus Disease 19 (COVID-19) is a new pandemic, declared a public health emergency by the World Health Organization, which could have negative consequences for pregnant and postpartum women [1]. Although pregnant women do not seem to be more likely to contract the infection than the general population [2–4], obstetric complications have been detected in infected pregnant women, including the threat of premature delivery, caesarean delivery and maternal complications in the postpartum period [3, 5]. Antenatal or intrapartum vertical transmission (transmission from a woman to her newborn) might also be possible and can result in severe consequences for the newborn [6, 7].

In light of the unprecedented crisis brought about by the COVID-19 pandemic, many perinatal healthcare practices have been changed in an attempt to reduce the risk of transmission of the novel coronavirus. These include cancelation of prenatal and postpartum courses routinely provided to mothers, adjustments to prenatal and postpartum appointments, restrictions to the partner’s presence during childbirth and postpartum visitation and, in some cases, face-to-face consultations replaced by teleconsultations [8, 9]. At the same time, the confinement and social distancing measures implemented to contain the spread of COVID-19 have been particularly challenging for women during the perinatal period [10]. The lack of information about this new virus, the social isolation and perceived loneliness, women’s worries over their own health and fear of infection and transmission of the virus to their fetus/newborn, together with sudden changes in maternity-care practices, may result in increased psychological distress during the perinatal period [11].

The perinatal period (from pregnancy to 1 year after childbirth) is, in general, a vulnerable time for the acute onset and recurrence of mental disorders. According to pre-pandemic data, it is estimated that 1 in 5 women will develop a mental disorder in the perinatal period [12–15]. Perinatal depression is the most common disorder during pregnancy and the postpartum period and carries long-lasting adverse effects for women, their partners and their infants, while also imposing a strong burden on their families and society as a whole [16]. The scarce evidence published to date suggests that mental disorders in pregnant women have increased since the COVID-19 outbreak. Studies conducted in Canada [17], China [18], Turkey [19, 20] and Italy [21] report that the rates of depression and anxiety in pregnant women more than doubled compared to studies prior to the pandemic. However, the few studies published so far have some limitations, such as a cross-sectional design, small sample sizes, the absence of assessment throughout the postpartum period or the omission of important factors for the understanding of perinatal mental health, including governmental restriction measures, healthcare practices implemented at the maternity hospitals and women’s coping strategies. Thus, further research using prospective comprehensive assessments, examining women’s psychological distress, as well as potential risk and protective factors, is needed in order to provide an evidence-based foundation for the understanding of the impact of the COVID-19 pandemic on women’s perinatal mental health [11, 22].

In order to investigate and recommend best practices that may mitigate the impact of the COVID-19 pandemic on women’s mental health [11, 23], members of the “Research Innovation and Sustainable Pan-European Network in Peripartum Depression Disorder – Riseup-PPD” (Cost Action 18138), funded by the Horizon 2020 Framework Programme of the European Union, established the “Perinatal Mental Health and COVID-19 Pandemic” Task Force. Currently, the research team is conducting an international prospective cohort study aimed at filling the gaps in research on the impact of the COVID-19 pandemic on perinatal mental health (Riseup-PPD-COVID-19), and this article presents the study protocol.

## Methods

### Aim

The main aim of this prospective study is to investigate the impact of the COVID-19 pandemic on perinatal mental health (depression, anxiety, and post-traumatic stress symptoms).

The primary objectives are the following:

1. To evaluate changes in perinatal mental health outcomes (symptoms and indices of clinical risk for mental disorders) during the COVID-19 pandemic.

We will evaluate the changes in (a) perinatal mental health symptoms (depression, anxiety and post-traumatic stress disorder [PTSD]); and (b) clinical risk indices (proportion of women above the clinical cut-off point on a validated self-report scale) for depression and anxiety disorders. We will compare the results to similar pre-pandemic studies in all the countries. We will also assess the acute and long-term mental health impact of different phases of the COVID-19 pandemic by measuring the changes in perinatal mental health outcomes between the initial survey and the follow-up assessments.

2) To determine the risk and protective factors that predict perinatal mental health outcomes during the COVID-19 pandemic.

We will determine the risk and protective factors that predict PTSD symptoms and the clinical risk of depression and anxiety disorders (proportion of new cases above the cut-off point on validated self-report scales in the follow-up period) during the COVID-19 pandemic. Factors included in our study were based on recent literature [24–28] and include demographic variables, COVID-19 epidemiology and public health measures, perinatal experiences during the COVID-19 pandemic, and psychological distress.

Furthermore, additional secondary objectives related to the impact of COVID-19 on perinatal mental health will also be examined: the comparison of perinatal mental health outcomes between the countries participating in the study; breastfeeding and its protective role in perinatal mental disorders; the risk of perinatal mental disorders in COVID-19-positive women vs. COVID-19-non-positive women; and factors associated with the level of distress of women during the perinatal period.

We hypothesize an increase in negative perinatal mental health outcomes (symptoms and clinical risk) among women during the COVID-19 pandemic. We also hypothesize that common risk factors (e.g., low income, low social support, high level of psychological distress, previous history of mental disorders) will be associated with an increase in the clinical risk of depression and anxiety during the COVID-19 pandemic. Additionally, we hypothesize that pandemic-related risk factors, such as being exposed to and/or having a positive diagnosis for COVID-19, will be associated with increased risk of perinatal mental disorders.

### Study design

This is an international prospective observational cohort study, with a baseline and three follow-up assessments: at one, three, and six months post-baseline. The observation period will be 6 months, from the initial evaluation to the last follow-up assessment. The overall study will span from 15 June 2020 to 15 June 2021.

### Setting

This international study will be carried out in 11 European countries (Albania, Bulgaria, Cyprus, France, Greece, Israel, Malta, Portugal, Spain, Turkey and the United Kingdom), Argentina, Brazil and Chile.

### Participants

The study population consists of women during the perinatal period. The rationale is to evaluate women throughout their pregnancy and during 1 year after childbirth, for a six-month period (from baseline to follow-up). Women can, thus, enter the study during pregnancy or at any time during the first 6 months following childbirth [29].

The inclusion criteria at baseline are:

- Being pregnant or a biological mother of a child six months of age or younger
- Women 18 years of age or older
- Living in one of the countries involved in the study
- Consenting to participate in the study

The exclusion criteria are:

- Not being currently pregnant or not being the biological mother of a child six months of age or younger
- Women younger than 18 years of age
- Living in a country that does not participate in the study.
- Not consenting to participate in the study

### Sample size

We have not set any restrictions on participant enrollment. However, a representative sample size was calculated according to the number of newborns in the previous year in each country. Thus, we estimated a minimum sample size of 300 participants per country, based on an α-level of 0.05 and heterogeneity equal to 50%.

### Variables and measures

The variables and measures planned for the different phases of the study are presented in Table 1.

**Table 1:** Variables and measures used in each phase of the study

Variables	Measures	Baseline	T1	T2	T3
*Independent/predictor*					
COVID-19 epidemiology and public health measures	OxCGRT	X	X	X	X
Demographic variables	14 questions	X			
Perinatal health care experiences related with COVID-19	COPE-IS	X			
COVID-19 exposures and symptoms	COPE-IS; COPE-IU	X	X	X	X
COVID-19 financial impact	COPE-IS; COPE-CF	X	X	X	X
COVID-19 social support impact	COPE IS; COPE-CF	X	X	X	X
COVID-19 coping strategies	COPE-IS; COPE-IU	X	X	X	X
COVID-19 emotional impact	COPE-IS; COPE-IU	X	X	X	X
Health background, mental health, and substance use	COPE-IS	X			
Psychological distress	BSI-18	X	X	X	X
*Dependent/outcome*					
Depression symptoms	EPDS	X	X	X	X
Anxiety symptoms	GAD-7	X	X	X	X
PTSD symptoms	10 questions from PTSD Checklist for DSM-5 included in COPE-IS; COPE-IU	X	X	X	X

T1: 1-month follow-up; T2: 3-month follow-up; T3: 6-month follow-up; PTSD: Post-traumatic Stress Disorder; OxCGRT: Oxford COVID-19 Government Response Tracker; COPE-IS: Coronavirus Perinatal Experiences - Impact Survey; COPE-IU: Coronavirus Perinatal Experiences Scale - Impact Update; COPE-CF: Coronavirus Perinatal Experiences Scale - Care Follow Up; BSI-18: Brief Symptom Inventory-18; EPDS: Edinburgh Postnatal Depression Scale; GAD-7: Generalized Anxiety Disorder Screener

### Independent/predictor measures

#### Oxford COVID-19 government response tracker (OxCGRT)

We will collect the following macro variables related to the COVID-19 epidemiological data and public health measures publicly available for each of the participating countries from the Oxford COVID-19 Government Response Tracker (OxCGRT) [30]: (1) Population; (2) Number of confirmed deaths; (3) Number of confirmed COVID-19 cases; (4) Case fatality rate; (5) Government Stringency Index [30], a composite score from 0 to 100 that considers the strictness of containment strategies (closures, movement restrictions) and information campaigns; (6) Containment and Health Index [30], a composite measure which combines ‘lockdown’ restrictions and closures with measures such as testing policy and contact tracing, short term investment in healthcare, as well investments in vaccines; and (7) Stay-at-home requirements or household lockdowns, which include: (a) No measures; (b) Recommended not to leave the house; (c) Required to not leave the house with exceptions for daily exercise, grocery shopping, and ‘essential’ trips; (d) Required to not leave the house with minimal exceptions (e.g., allowed to leave only once every few days, or only one person can leave at a time, etc.). These data will be extracted according to the date of the baseline and subsequent follow-up analysis.

#### Demographics

This questionnaire will include a subset of 14 questions about the women’s date and country of birth; state/city of residence; educational level; number of previous pregnancies; number of biological children (including stillbirths); number of people living at home (adults and children); marital status; cohabitation with partner; characteristics of the home (inside and outside spaces in square meters); and living environment changes since the beginning of the pandemic.

#### Coronavirus perinatal experiences (COPE) questionnaires

In order to assess the experiences of the women during the COVID-19 pandemic, a modified version of the Coronavirus Perinatal Experiences - Impact Survey (COPE-IS) [31] will be administered at the baseline assessment. The questionnaire assesses several areas of impact: perinatal experiences related to the COVID-19 pandemic; exposure and symptoms; financial impact; social support impact; social distancing and activity restrictions; coping and adjustment; emotional impact; physical and mental health history and substance use.

In the follow-up assessments, the women’s experiences will be measured using a modified version of the Coronavirus Perinatal Experiences Scale - Impact Update (COPE-IU) [32] and the Coronavirus Perinatal Experiences Scale - Care Follow Up (COPE-CF) [33]. The COPE-IU evaluates exposure to COVID-19, symptoms, impact on daily life, experiences and feelings, whereas the COPE-CF evaluates perceived support and care and the social and financial impact of the pandemic.

The sections included in the modified version of the COPE-IS [31], COPE-IU [32]and COPE-CF [33]are described below.

##### Perinatal health care experiences related to COVID-19

Twenty-six items regarding parity, health problems during pregnancy, type of pregnancy, prenatal and postpartum care support and resources available during this period, changes experienced as a result of the COVID-19 outbreak, and possible concerns about changes in family/friend support, medical care during childbirth and the child’s health. For postpartum women specifically, the survey also includes questions about the infant’s place of birth, breastfeeding, changes experienced in birth plans and level of distress experienced related to changes in birth and postpartum plans [31].

##### COVID-19 exposures and symptoms

Six items about (1) the diagnosis of COVID-19; (2) any symptoms compatible with the coronavirus disease; (3) contact with someone who has been diagnosed with COVID-19; (4) any death of family or friends due to COVID-19; (5) the level of distress about COVID-19-related symptoms or potential illness: (5a) their own illness or (5b) in their friends and family [31, 32].

##### COVID-19 financial impact

Two items about (1) the current employment situation and (2) the financial impact of the COVID-19 outbreak [31, 33].

##### COVID-19 social support impact

Six items about (1) seeking support, (2) support provided, (3) need for support, (4) perceived support; (5) level of distress experienced with disruptions to social support due to the COVID-19 pandemic; and (6) number of weeks participants have been observing the lockdown guidance [31, 33].

##### COVID-19 coping strategies

A total of 23 items regarding potential coping strategies (problem-focused or emotion-focused) to cope with the stress related to the COVID-19 pandemic (for example, “getting a good night’s sleep”; “talking with friends”). The participants must indicate all the strategies that they are implementing [31, 32].

##### COVID-19 emotional impact

Four items assessing the subjective impact of the COVID-19 pandemic. The questions include: (1) overall level of impact on daily life due to the COVID-19 pandemic; (2) overall level of stress related to the COVID-19 outbreak; (3) perceived positive or negative impact of the COVID-19 pandemic; and (4) how long participants think it will take for things to go “back to normal”? [31, 32].

##### Physical and mental health history and substance use

Thirteen items addressing: 1) the women’s and family (partner, child or other members of the household) history of the following medical conditions: respiratory problems, diabetes, heart disease or hypertension, lung disease; liver disease; cancer; disease compromising the immune system; mood and/or anxiety disorders; 2) whether currently receiving treatment for mental disorders (for example, depression, anxiety, stress, ADHD, bipolar disorder, eating disorder or PTSD); 3) current substance use; and 4) current treatment of medical conditions [31].

#### Brief symptom Inventory-18

The Brief Symptom Inventory-18 (BSI-18), omitting suicidality, will be used to evaluate psychological distress [34] in the last week. The BSI-18 is an 18-item self-report questionnaire which includes a summary scale of overall distress, the Global Severity Index (GSI), and three subscales: depression, anxiety, and somatization. Each item is rated on a 5-point scale, with distress ratings ranging from 0 (not at all) to 4 (extremely). For the purposes of the current study, only the GSI will be examined. The GSI level is the summed score of all the items [34].

#### Dependent/outcome measures

##### Edinburgh postnatal depression scale

The Edinburgh Postnatal Depression Scale (EPDS) [35] is the most widely used self-report scale designed to identify women at risk for perinatal depression [36]. The 10-item scale indicates how women felt during the previous week. The items assess symptoms of sadness, anxiety and thoughts about death. Scores range from 0 to 30. The depression level is the sum score of all the items, and higher scores indicate greater symptom severity. A threshold of 13 or more will be used to identify clinically significant symptoms [37]. Although a number of cut-off points have been used in different populations [38], maximum accuracy with a probable diagnosis of depressive disorder has been shown at this cut-off point in reviews of international EPDS validation studies [36–39].

#### Generalized anxiety disorder screener

Symptoms of anxiety will be evaluated using the validated self-report questionnaire Generalized Anxiety Disorder Screener (GAD-7) [40], which is based on criteria from the Diagnostic and Statistical Manual of Mental Disorders (DSM)-IV and DSM-IV-TR [41]. Even though GAD-7 was developed for generalized anxiety disorder, it is also used as a measure for anxiety for research purposes [42, 43] and clinical management for antenatal and postpartum mental health [44]. The seven items assess worry, tension, restlessness, and irritability. The GAD-7 total score ranges from 0 to 21. The anxiety level is the sum score of all the items, with higher scores reflecting greater anxiety severity. We will use a threshold of 10 or more points for a participant to be considered as belonging to a clinical range [14, 40]. The GAD-7 has shown good reliability and construct validity in pregnancy and the postpartum period [40, 45, 46].

#### Post-traumatic stress disorder (PTSD) checklist

Post-traumatic stress symptoms associated with COVID-19 will be evaluated via a subset of 10 questions from the Post-Traumatic Stress Disorder (PTSD) checklist for DSM-5 [47, 48]. These 10 questions ask participants to indicate the extent to which they experienced: (1) Feeling super alert or watchful or on guard; (2) Feeling jumpy or easily startled; (3) Having difficulty concentrating; (4) Trouble experiencing positive feelings; (5) Feeling guilty or blaming yourself; (6) Feeling irritable, angry or aggressive; (7) Repeated disturbing and unwanted thoughts about the COVID-19 outbreak; (8) Repeated disturbing dreams about the COVID-19 outbreak; (9) Trying to avoid information or reminders about the COVID-19 outbreak; and (10) Taking too many risks or doing things that could cause you harm. All items refer to the previous seven days and are answered on a five-point Likert scale, ranging from 0 (Not at all) to 4 (Extremely) with possible total scores ranging from 0 to 40. Higher scores indicate greater symptom severity. The PTSD symptom level is a summed score of all the items [47]. The subset of questions from the PTSD checklist are included in the COPE-IS and COPE-IU questionnaires [31, 32].

### Validation of the questionnaires

We will use available validated versions of the EPDS, GAD-7 and PTSD DSM-5 checklist in the official language of all the participating countries. However, COPE-IS, COPE-IU and COPE-CF are newly developed measures, and psychometric properties are yet to be established. Scoring procedures also need to be determined. The COPE questionnaires were originally available in English and translated to Spanish and German. Thus, researchers from each country involved in the study performed the translation and cultural adaptation of the questionnaires from English into the official language of their country, following several methodological steps, defined a priori, as detailed in Table 2. No major discrepancy was identified in any of the cross-cultural adaptations.

**Table 2:** Cross cultural adaptation of the COPE questionnaires

• Adaptation of “demographic variables”	
• Forward and backward translation from the original version	
• A pilot study with a small (*N* ≥ 4) sample of pregnant women and new mothers (to assure comprehensibility of the items as intended)	
• Revision of the instrument by an expert committee (in the perinatal field) in each country (*N* ≥ 2 persons)	

### Ethical standards

This study is conducted according to the principles expressed in the Declaration of Helsinki. Ethical approval was obtained from all the countries before starting the project. Handling of the study data will comply with all the national required standards for data protection. Electronic informed consent will be obtained from all the participants, and the confidentiality of all the information provided will be ensured.

Some survey questions can elicit changes in the emotional state of participants. In order to address this issue, a debriefing procedure will be made available; at the end of the survey, a list of up-to-date services and resources will be provided for each country covering a range of needs, from information about the COVID-19 pandemic to the general mental health and perinatal care services available in each country. Additionally, contact information for the lead research team for each country will be given in case participants would like to request additional information. The researchers will publish the results of the study on the website and will send information about the global results of the study to the participants upon request.

### Data collection

Participants will be recruited through social media advertising (Twitter, Facebook, Instagram, LinkedIn, ResearchGate, WhatsApp, etc.), networks of organizations (including universities, health centers, NGOs working in the field of perinatal mental health), policymakers, local organizations, and other stakeholders (using the network provided by the Riseup-PPD), as well as personal networks of colleagues and acquaintances of the research team members, and direct contact by mobile message or email.

Participants will be asked to click on the project website link (see the general project website: https://momsduringcovid.org) to be directed to the online questionnaire for their specific country. First, they will read an electronic consent form presenting an overview of the study aims, content of the questions asked, potential risks and benefits, and ethical aspects of the study (i.e., voluntary participation, confidentiality and secure storage of the data, and absence of any type of compensation). At the bottom of the form, they will be asked to confirm a set of eligibility criteria and to provide their consent to access the study. Participants who do not meet the predefined inclusion criteria will be directed to a message thanking them for their interest and informing them of the required eligibility criteria for participation in the study.

The baseline questionnaire is estimated to take approximately 20 min to complete. Eligible participants who complete the baseline questionnaire will be invited to provide their email or phone number to be contacted to participate in the three follow-up assessments. Participation in the follow-ups will be optional and participants will need to provide their consent to be contacted by email or phone (text messages only). Attached to this request, we will provide a clear plan to guarantee confidentiality and protection of the contact information provided (this information will also be provided in the Informed Consent).

### Data analysis

Patterns of missingness in the data will be studied and the Expectation-Maximization algorithm will be implemented to impute missing data. Attrition analyses will be carried out to examine differences between participants who completed all the assessments and those who did not. Descriptive statistics will be examined for the study variables for all the assessment times. Measurement equivalence will be studied in order to ensure we are measuring the same variables across countries.

Changes in mental health between baseline and the follow-ups will be examined by testing latent growth curve models for each outcome. Moreover, the relationships between the rates of change in predictors (i.e., risk and protective factors) and mental health results during the follow-up will be analyzed. Multilevel analyses will be performed to control the moderation of demographic variables in those interrelations. Regarding the overall fit of these models, Satorra-Bentler χ^2^, CFI, RMSEA and the confidence interval for the RMSEA statistics will be calculated. Lagrange multipliers and the Wald test will be performed sequentially to improve the fitness of the models, following the hypotheses of the study. Standardized coefficients and measurement equations with scores in R^2^ will be reported. These analyses will be undertaken using the statistical program EQS. 6.3.

To determine the risk and protective factors to predict perinatal mental health outcomes during the COVID-19 pandemic, a random sample of 70% of the data will be used to derive the machine learning algorithms including regularized logistic regression, random forest, decision tree, and gradient boosting to predict the risk of PPD, and their performance, and the remaining 30% for validation. First, in the derivation sample, all the predictors described above will be entered into the models to estimate the probability of depression or anxiety or PTSD. Beginning with a model containing all potential covariates, the variable with the least significant *P* value will be removed and tested using the likelihood-ratio test until all variables left in the model significantly (at alpha = 0.05) contribute to the model.

In regularized logistic regression models, results will be presented as odds ratio (OR) with 95% confidence intervals (CIs). The R^2^ will be used to calculate the proportion of the explained variance of clinical outcomes by the selected predictors. The different aspects of model performance will be studied, including calibration and discrimination. Calibration will be assessed using the Brier score and by plotting the non-parametric estimate of the association between the observed frequencies and the predicted probabilities. The receiver operating characteristic (ROC) curves (and the corresponding area under the ROC curve—AUC) will be calculated to test for discrimination. To correct optimism, internal validation will be performed for each model using the bootstrap procedure with 500 bootstrapped samples. The final models will be selected to derive scores for clinical use, and nomograms will be created. Criteria for this selection will include both discriminant ability (defined by the AUC) and model simplicity. Finally, the coefficients (scores) derived from the derivation cohort will also be validated in the validation cohort. These statistical analyses will be carried out in R and Python. The analyses will conform to the reporting standards of transparent reporting of a multivariable prediction model for individual prognosis or diagnosis (TRIPOD) [49].

### Dissemination and data sharing plan

To enhance data reporting transparency, this study will be reported in accordance with the Strengthening the Reporting of Observational Studies in Epidemiology (STROBE) Statement: Guidelines for Reporting Observational Studies) [50].

Data and resources will be shared with other eligible investigators through academically established means. The datasets used and/or analyzed during the study will be available upon reasonable request. The results from this work will be published as full-length, peer-reviewed manuscripts and presented at national and international conferences.

## Discussion

Several studies on COVID-19 and pregnancy have been recently published, but the impact of this pandemic on perinatal mental health is yet to be properly evaluated. Currently, no international prospective cohort study has been published on the impact of COVID-19 on perinatal mental health [51].

An international collaboration and global perspective will enrich the understanding of the impact of COVID-19 on perinatal mental health, while examining potential similarities and differences across the countries involved. The current investigation aims to offer new insights into this new research domain. Further, it will address some of the existing gaps in this field: (1) the international nature of the study will enhance the external validity of the findings by using multi-site comparable instruments; (2) the governmental restriction measures during the COVID-19 pandemic (e.g. Government Stringency Index; Containment and Health Index) will be extracted from the Oxford COVID-19 Government Response Tracker (OxCGRT) [30] dataset; (3) the participants will be women during the perinatal period; (4) we will assess healthcare practices and potential risk and protective factors cited in the literature as relevant for perinatal mental disorders; and (5) the study will include four assessments (baseline and three follow-ups) during a six-month period. This will enable us to address the impact of the different countries’ governmental restriction measures on the perinatal mental health outcomes, to identify the variability in risk and protective factors over time, and to examine their association with mental health outcomes. Furthermore, we will develop predictive models for perinatal mental health outcomes using machine learning approaches [52–54].

The present study will also have some limitations. First, our sample will not be a random probability sample, since there was a need to rapidly obtain a sample of perinatal women, so non-purposive sampling was used. Second, using online surveys limits the participation of some important groups in the population, including those women with no access to internet or social media, low educational level (limiting comprehension of the questions), or not fluent in the official language of the country of residence. It is also possible that the survey attracts participants interested in the topic and affected by psychological factors (e.g., need for gratification, personality dispositions), creating the possibility of a sampling bias [55]. Last, all perinatal mental health outcomes will be evaluated using valid self-report screening instruments instead of clinician administered interviews, which may result in over- or under-estimation of perinatal mental health disorder prevalence and incidence.

### Significance and practical implications of the study

A previous position paper highlights that an immediate public health priority is evidence-based research on the mental health effects of the COVID-19 pandemic in vulnerable populations [22], such as women during the perinatal period [11]. Furthermore, there is an urgent need for research to address how to mitigate the mental health consequences of COVID-19 in women during the perinatal period [22].

The findings will provide critical information regarding the risk and protective factors for perinatal mental health disorders during the COVID-19 pandemic. The results of this study will offer clarification on the impact of the pandemic on women during the perinatal period, allowing the development of comprehensive models for the prediction of perinatal mental disorders, specifying the contribution of each risk and protective factor and contributing to the design of tailored interventions to support women during the COVID-19 pandemic. This will allow perinatal healthcare providers to assess women at risk of developing mental health disorders during the pandemic and provide early intervention as well as develop preventive measures. In addition, the findings of the study will assist policymakers and healthcare providers in developing guidelines and preventive interventions for perinatal mental disorders in future pandemics.

## Conclusion

In conclusion, this international prospective cohort study aims to robustly/longitudinally assess the impact of the COVID-19 pandemic on women’s perinatal mental health. This information will provide vital understanding of the impact of the pandemic on perinatal mental health, which may aid in the implementation of tailored interventions and provide empirical evidence for policymakers on future decisions in similar scenarios as well as on future planning of interventions.

## Data Availability

Not applicable.

## Abbreviation

COVID-19: Corona Virus Disease 19
